# Effect of High Pressure, Calcium Chloride and ZnO-Ag Nanoparticles on Beef Color and Shear Stress

**DOI:** 10.3390/foods9020179

**Published:** 2020-02-12

**Authors:** Begoña Panea, Pere Albertí, Guillermo Ripoll

**Affiliations:** 1 Animal Production and Health Unit, Centro de Investigación y Tecnología Agroalimentaria de Aragón, Avda. Montañana, 930, 50059 Zaragoza, Spain; palberti@aragon.es (P.A.); gripoll@aragon.es (G.R.); 2 Instituto Agroalimentario de Aragón-IA2 (CITA-Universidad de Zaragoza) Avda. Montañana, 930, 50059 Zaragoza, Spain

**Keywords:** pressure, marination, color, texture, packaging

## Abstract

This study investigates how the use of a combination of high-pressure treatment, steak marination and active packaging influences beef color and shear stress. A 2 × 2 × 2 × 3 factorial design was applied, including pressure, marination, packaging and storage time. Many significant interactions among factors were found, but the effects of pressure and marination were so high that the effect of packaging was almost undetectable. Independent of storage type, pressurized treatments presented higher values for both L* and *h_ab_* than unpressurized treatments, and independent of pressure application, the increase in L* and *h_ab_* with storage time was higher for marinated treatments than for unmarinated treatments. In unpressurized samples, marination provoked an increase in L*, a* and *h_ab_* and a decrease in $C_{ab}^{*}$, whereas in pressurized samples, marination had no effect on color. Pressurized samples always showed higher values for shear stress (on average 71% higher) than unpressurized samples.

## 1. Introduction

The high-pressure technique is applied in the food industry mainly to inactivate microbial growth, resulting in safety and extending the shelf life of products [1,2], but it can also be used to improve meat tenderness. Nevertheless, although the use of high pressure in meat tenderization is well known [2,3,4], its implementation in industry has been limited because the effect of high pressure on meat texture depends on several factors, such as the pressure applied, temperature, time, muscle and aging period. In addition, the application of high pressure promotes intense decoloration [1], which can cause consumer rejection since color is one of the most important factors in buying decisions [5]. In addition, high-pressure is an expensive technique since the machine costs about 500.000 euros and, in addition, 0.10–0.15 euros per sample (provider data). 

These changes in color are mainly due to two processes: adiabatic heating of the meat [6] and changes in the oxygen consumption rate [7]. Lower oxygen consumption rates allow greater penetration of oxygen into the muscle, resulting in more stable color [7]. Thus, the use of high-oxygen packaging could aid in preserving underpressurized meat color. Nevertheless, when meat is packed in high-oxygen packaging, it is more susceptible to lipid and protein oxidation, which in turn also causes changes in color and sensory meat quality. The use of active packaging could be a solution, and, in this sense, nanotechnology has broken into the packaging industry in recent years [8]. Metals and their oxides, such as ZnO, TiO_2_, MgO and CaO, are particularly interesting because they are safe for animals and humans [9], and they are allowed in the U.S. Food and Drug Administration list [10] as well as in European Regulation R.450/2009 [11]. Among metal cations, silver ions are known to have the highest antimicrobial capacity against a wide spectrum of Gram+ and Gram– microorganisms, but ZnO has gained interest due to its low cost [12].

Another increasingly applied technique to counter the effect of high pressure is the use of marinades. Marinating enhances meat flavor, juiciness and tenderness and is; therefore, especially interesting for use in low-quality muscles, such as *semitendinosus* [13]. Unfortunately, depending on the marinade liquid, it can also lead to color and texture modifications [14]. Thus, the sole use of marinades may not be enough to counter pressure effects.

The aim of the present paper was to investigate how the use of a combination of high-pressure treatment, steak marination and active packaging influences beef color and shear stress. In the literature, studies combining high pressure and marination [15] or packaging and marination [16] can be found, but to the best of our knowledge, this triple combination of pressure, marination and packaging has not been previously investigated.

## 2. Materials and Methods

### 2.1. Packaging: Production Method, Chemical Composition and Migration Assays

Packaging composed of LDPE (low-density polyethylene, LD 654, ExxonMobil, Chemical, Baytown, TX, USA) blended with a nano-antimicrobial master batch containing Ag and ZnO nanoparticles (Avanzare, Navarrete, Spain) at 0% and 5% *w*/*w* was produced. Details of the packaging production, composition and characteristics, as well as the migration assays, can be found in Panea et al. [17].

### 2.2. Meat Sampling and pH Measurement

Eight commercial carcasses from young bulls were used. The animals were slaughtered in a commercial abattoir at 13 months of age, had a cold carcass weight of 361 ± 37.0 kg, and were classified as U2 following the European Classification System [18]. Carcasses were kept at 4 °C until the 5th day post-mortem. Then, the *semitendinosus* muscle of the left half of the carcasses was excised and transported to the laboratory, and the pH was measured with a pH meter equipped with a Crison 507 penetrating electrode (Crison Instruments S.A., Barcelona, Spain).

A 2 × 2 × 2 × 3 factorial design was applied, including pressure, marination, packaging and storage time.

From each muscle, eight steaks (3 cm-thick) were chopped perpendicularly to the fiber direction, vacuum packed (MCOEX material bags, Coimbra Pack, S.L., Zaragoza, Spain) and kept at 4 °C for 24 h. Then, half of the steaks (named P) were treated with a high pressure of 600 MPa for 6 min, with water at 12 °C as the transmission fluid, using a Hyperbaric 6000 machine (Hiperbaric, S.A., Burgos, Spain), whereas the other half were not pressurized. Afterwards, all of the samples were removed from the bags and the color was measured (experimental day 0). Subsequently, both pressurized (P) and non-pressurized samples were split again, half placed into an LDPE-5%-nanoparticle tray (named N), half into LDPE-0%-nanoparticle trays. Next, half of both the LDPE-5% or LDPE-0%-nanoparticle trays were supplemented with calcium chloride solution to marinate the steaks (named M), the other half remained without marinade liquid. The m-calpain, responsible for the meat tenderization, needs a concentration of Ca^2+^ for activation between 1 and 5 nM [19]. However, when concentration of calcium chloride solutions increased from 0.1 to 0.3 M, meat became darker and more prone to oxidation [20]. Therefore, the concentration of the calcium chloride was set to 0.1 M. Then, all of the trays were wrapped with a PE-LD oxygen-permeable film (Coimbra Pack, S.L., Zaragoza, Spain), without contact with the meat surface, and kept at 4 °C for 6 or 13 days, which was considered time enough to see an effect, if one exists. 

Resuming, eight experimental batches were prepared:

- C: Control (unpressurized-LPDE 0%-unmarinated)
- N: unpressurized-LPDE 5%-unmarinated
- M: unpressurized-LPDE 0%-marinated
- NM: unpressurized-LPDE 5%-marinated
- P: pressurized-LPDE 0%-unmarinated
- PN: pressurized-LPDE 5%-unmarinated
- PM: pressurized-LPDE 0%-marinated
- PNM: pressurized-LPDE 5%-marinated.

**Figure 1:**
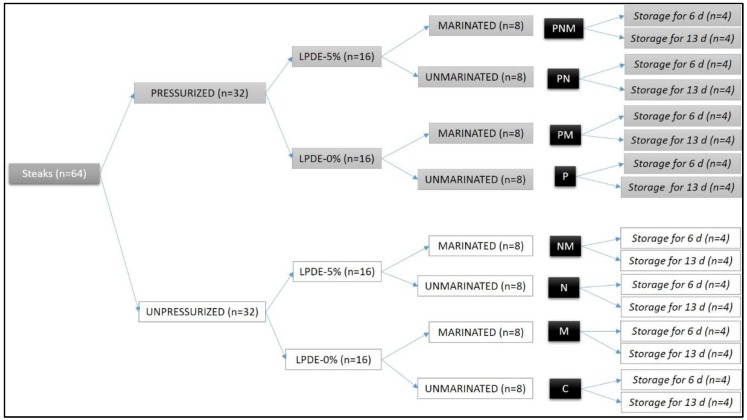
Experimental design.

The experimental design is shown in Figure 1.

### 2.3. Instrumental Procedures

#### 2.3.1. Color

Color was measured on the days 0 and 6 of treatment. Color changes were so evident at day 6 that to measure it at day 13 was considered unnecessary. All the samples used for color analysis were allowed to bloom for 90 min. The color was measured with a Minolta CM-2006d spectrophotometer (Konica Minolta Holdings, Inc, Osaka, Japan) in CIELAB space (CIE, 1986) with a measured area diameter of 8 mm. The specular component included 0% UV; the standard illuminant was D65, which simulates daylight (color temperature 6504 K); a 10° observer angle was used; and zero and white calibrations were applied.

The lightness (L*), redness (a*) and yellowness (b*) were recorded, and the hue angle (H°) and chroma (C*) indexes were calculated as $h_{ab}=\tan^{-1}\left(\frac{b*}{a*}\right)\cdot \frac{180{}^{\circ}}{\pi }$, expressed in degrees, and $C_{ab}^{*}=\sqrt{{\left(a*\right)}^{2}+{\left(b*\right)}^{2}}$. The relative contents of metmyoglobin (MMb) and oxymyoglobin (MbO_2_) were estimated by the ratios K/S_572/525_ [21,22] and K/S_610/525_, respectively [21,23]. These ratios decrease when the pigment content increases. The Kubelka-Munk K/S values were calculated using SpectraMagic NX (Minolta Co., Ltd., Osaka, Japan), and K/S at 572 and 525 nm were calculated by linear interpolation. Additionally, the ratio of light reflectance at 630 and 580 nm (R_630_/R_580_) [24,25] was calculated. Finally, the color difference between two stimuli (ΔE) was calculated as $\Delta E*=\sqrt{{\left(\Delta L*\right)}^{2}+{\left(\Delta a*\right)}^{2}+{\left(\Delta b*\right)}^{2}}$, only in the cases in which it was necessary to explain human eye-detectible differences [25].

#### 2.3.2. Texture

Samples of the eight muscles were evenly distributed according to experimental treatments and times. Because the size of the *semitendinosus* muscle was not enough to measure texture at three storage points (0, 6, and 13 days), it was decided to avoid the initial point. Therefore, it was assumed that differences in texture between treatments at day 0 would be unnoticeable and, consequently, texture was measured only on days 6 and 13 of treatment. Samples were vacuum packed and heated in a 75 °C water bath to an internal temperature of 70 °C, which was monitored with a Testo thermocouple equipped with a probe (Testo SE & Co. KGaA, Lenzkirch, Germany). A minimum of 10 subsamples with a 10 × 10 mm^2^ cross-section were obtained following a longitudinal configuration [26]. Samples were sheared using an Instron 5543 (ITW Test and Measurements, Essligen, Germany) fitted with a Warner–Bratzler device. The shear maximum stress (load at maximum peak shear force per unit of cross-section, in N/cm^2^) and toughness (amount of energy necessary to break the sample, in N/cm2) were recorded.

#### 2.3.3. Statistics

Statistical analyses were performed with the XLSTAT statistical package v.3.05 (Addinsoft, USA). Student’s *t*-test was performed to study the differences in pH between batches. Two independent general linear model procedures were carried out for color and texture analysis, with pressure application (yes/no), packaging type (with/without nanoparticles), marinade immersion (yes/no), and storage time (0 or 6 days for color; 6 or 13 days for texture) as fixed effects. The means and standard errors of all the considered variables were calculated. The Duncan test was used to compare means, and the level of significance was *p* < 0.05.

## 3. Results

### 3.1. pH and Color

The global mean pH was 5.66 (standard error = 0.015, results not shown), and no differences were observed between samples (*p* > 0.05).

Table 1 shows the *p*-values for the effects of studied factors on color variables. All studied factors except packaging type (LPDE-0% or LPDE-5%) affected almost all the studied variables. In addition, many significant interactions among effects were found, including the interaction between pressure application and packaging type (P×N) on L*, a* and b*.

**Figure 2:**
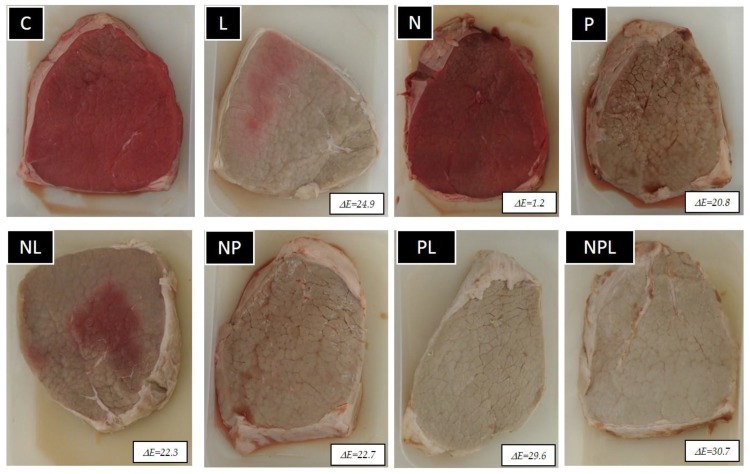
Steaks from the different experimental batches on day six of exposure and ΔE with respect to the control. C: Control (unpressurized-LPDE 0%-unmarinated); N: Unpressurized-LPDE 5%-unmarinated; M: Unpressurized-LPDE 0%-marinated; NM: Unpressurized-LPDE 5%-marinated; P: Pressurized-LPDE 0%-unmarinated; PN: Pressurized-LPDE 5%-unmarinated; PM: Pressurized-LPDE 0%-marinated; PNM: Pressurized-LPDE 5%-marinated.

The means of color variables as a function of treatment are given in Table 2 and a picture of the steaks are in Figure 2. The application of pressure resulted in an increase in L*, *h_ab_* and b* and a decrease in a* and metmyoglobin percentage. In unpressurized samples, marination provoked an increase in L*, a* and h_ab_ and a decrease in $C_{ab}^{*}$ without an effect on b*. Nevertheless, in pressurized samples, marination had no effect on color. Within a certain pressure–marinade combination, packaging type did not affect any of the color variables. In the C and N treatments, storage time only affected the metmyoglobin percentage, whereas in the rest of the treatments, all of the variables changed over time, except b*.

In Figure 2 are shown the ΔE values with respect to the control on day six of exposure. All of them, except the value of N batch, were higher than the 2.5–3 usually considered as a threshold for human eye detection [27,28]. 

**Figure 3:**
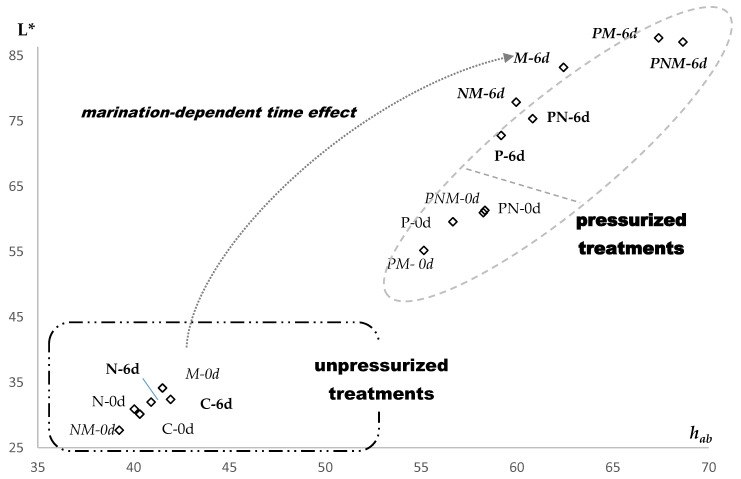
Beef color representation of L* and *h_ab_* as a function of the studied factors (pressure application, packaging type, marinate immersion and storage time). C: Control (unpressurized-LPDE 0%-unmarinated); N: Unpressurized-LPDE 5%-unmarinated; M: Unpressurized-LPDE 0%-marinated; NM: Unpressurized-LPDE 5%-marinated; P: Pressurized-LPDE 0%-unmarinated; PN: Pressurized-LPDE 5%-unmarinated; PM: Pressurized-LPDE 0%-marinated; PNM: Pressurized-LPDE 5%-marinated. Marinated treatments are in italics, and pressurized treatments are in bold.

Figure 3 is a representation of L* versus *h_ab_*. In all of the treatments except C and N, both L* and *h_ab_* increased with storage time. Independent of the packaging and storage type, the pressurized treatments (P, PN, PM and PNM) presented higher values for both L* and *h_ab_* than the unpressurized treatments (C, M, N, and NM); and independent of the pressure application and packaging type, the increase in L* and *h_ab_* with storage time was higher for the marinated treatments (M, NM, PM, and PNM) than for the unmarinated treatments (C, N, P, and PN).

### 3.2. Texture

Table 3 shows the *p*-values for the effects of the studied factors on texture variables, and Table 4 shows the means and standard errors of the texture variables. Only pressure application affected the texture variables, but a significant interaction was found between pressure and packaging type in terms of shear stress (*p* = 0.027). Thus, when samples were pressurized, packaging type influenced both the shear stress and toughness, whereas when samples were unpressurized, only shear stress was affected by the packaging type. Independent of the packaging type, pressurized samples always showed higher values for shear stress (on average, 71% higher) than unpressurized samples.

## 4. Discussion

### 4.1. Color

Values found for color variables were similar to those reported by other authors in *semitendinosus* muscle [7,15,16].

The effect of high pressure on meat color has been broadly reported. It is generally accepted that pressure treatment increases beef lightness, whereas redness decreases and yellowness remains more or less unchanged [1,3,15,29], in agreement with the current results. Because of these changes, pressurized meat shows a pink color similar to that of cooked meat [30]. Pressure application occurs via an adiabatic process that implies an increase in meat temperature [6] of approximately 3 °C per 100 MPa [31], which could explain why the general appearance of the meat resembled cooked meat instead of fresh meat.

These changes in color are associated with modifications in myoglobin structure, haem displacement, the formation of metmyoglobin, the denaturation of myofibrillar proteins and changes in connective tissue [3].

Regarding myoglobin, Buckow et al. [32] reported that the primary and secondary structures of globular proteins are rarely affected by high pressure because covalent bonds are minimally compressible, whereas the tertiary and quaternary structures are damaged by high pressure. In addition, pressure promotes the displacement of haem groups and iron ions, and, in the range of 250 to 500 MPa, the conversion of ferrous myoglobin to ferric metmyoglobin can be found, resulting in a greener color [32]. On the other hand, a linear relationship was observed between the K/S_572/525_ ratio and the amount of metmyoglobin accumulated on the steak surface, and it has been stated that consumers start to discriminate color changes when the percentage of metmyoglobin reaches 20% [7], that is, when the ratio value is above 1.20 [33,34]. In the current results, the K/S_572/525_ ratio ranged between 0.94 and 1.40; therefore, the percentage of metmyoglobin would range between 50% and 0%, with lower values for pressurized samples. Under the current conditions, it seems that the adiabatic increase in temperature and not the metmyoglobin content was responsible for the surface meat color.

In addition to these generally accepted overall color changes, it has been reported in literature that the pressure effect depends largely on treatment conditions. Then, the L* value increases even at low pressures (approximately 150 MPa), but no additional changes in L* value were observed for pressures higher than 350 MPa [32]. Increases in L* values have been related to protein denaturation [35], which affects light reflectance. On the other hand, the decrease in a* value is more evident above 400 MPa [32], and pressures higher than 300 MPa cause oxymyoglobin to transform into metmyoglobin [36]. The temperature at which the high pressure was applied also influenced the effect on meat discoloration. In general, the higher the temperature is, the higher the pressure effect [1]. For example, Marcos et al. [37] stated that the increase in L* value was higher when pressure was applied at 30 °C than when it was applied at 20 or 10 °C.

Regarding the effect of storage time, contradictory results can be found in the literature. Cheah and Ledward [38] reported that if pressure is applied in the first two days after slaughter, pressure treatment increases color stability during subsequent inspection, whereas if pressure treatment is applied several days after slaughter, it has no effect on color stability. In contrast, King et al. [39] reported a decrease in L*, a*, b* and $C_{ab}^{*}$* and an increase in *h_ab_* from the 0th to 9th day of exposure in *semitendinosus* muscle. The current results (Figure 2) show that color changes over time occurred independently of the pressure, although they were more marked when samples were pressurized.

The effect of marinating on meat color has been described by several authors. Cruzen et al. [16] reported that a calcium-salt marinade promotes an increase in L* values without affecting the a* values of *semitendinosus* muscle. In addition, these authors indicated that when meat was unmarinated, the L* values increased from the first day to the 9th day of storage, whereas in marinated samples, this evolution did not happen. Similarly, Klinhom et al. [40] reported an increase in L* values and a decrease in a* values when *semimembranosus* samples were marinated with a 0.2 M calcium chloride solution.

The lack of an effect of combined Ag-ZnO treatment on meat color was previously found in our laboratory in poultry [17]. In the current experiment, the effects of pressure and marination were so high that the effect of packaging was almost undetectable.

It can be seen in Figure 2 that differences between treatments are so evident that a consumer visual test, which would be interesting if the effect were less noticeable, was considered unnecessary.

### 4.2. Texture

Several mechanisms have been proposed to explain the effect of pressure on shear force. The reported mechanisms include the destruction of the sarcomere structure at the I-line, M-line and Z-line levels, the aggregation of fine and thick filaments [41], the unfolding of connective tissue, a decrease in protein solubility [15], the activation of autolytic activity, the release of calcium into cytosol and the denaturation of enzymes.

As occurs with color, the effect of pressure treatment on meat texture depends on the pressure, meat rigor state and temperature [3].

It has been described that moderate pressures (<300 MPa) results in meat tenderization, whereas medium or high pressures (>400 MPa) induces meat toughening, and increases in pressure to 800 MPa leads to small changes [6]. In a meta-analysis studying the effect of pressure on pork [3], it was found that pressures of 100–250 MPa resulted in a significant reduction in shear force of approximately 0.92 kg, whereas if the pressure was higher than 250 MPa, the reduction was only approximately 0.38 kg. Morton et al. [4] described that upon applying 175 MPa, shear force decreased by approximately 60% in *longissimus thoracis* muscle and approximately 43% in *gluteus medius* muscle. The shear force reduction is higher when pressure is applied in a pre-rigor state than when it is applied in a post-rigor state [31]. Pressures of approximately 100–200 MPa cause the trickling of cathepsins and calcium into the cytosol and a decrease in calpastatin. As a result, there is a disruption of the myofibrillar structure and an improvement in tenderness [6,31,42]. Pressures above 300 MPa induce the contraction of sarcomeres and the denaturation and fragmentation of proteins, but there is also an increase in the area of the myofibrils, resulting in toughening of the meat, in agreement with the current results [41,43].

The sensitivity of proteins to pressure is temperature dependent, and it was much higher at temperatures of approximately 60–70 °C than at 20 °C [6,42,44]. A combination of low pressure (less than 200 MPa) and high temperature (approximately 60 °C) results in meat tenderization because enzymes are active in these conditions, allowing proteolysis. In addition, at 60 °C, there was collagen denaturation [31].

Ueno et al. [45] reported that treatment with 100–400 MPa at 4 °C for 5 min caused deformation of the endomysium, whereas other authors reported a reduction of the thermal stability of collagen and a separation of the perimysium when meat was pressurized above 200 MPa at room temperature [46,47]. Nevertheless, under our experimental conditions (12 °C, 600 MPa), neither enzyme activity nor collagen denaturation was allowed, and pressurized samples were tougher than unpressurized samples.

Several authors [40,48,49] have reported that calcium–salt marinades promote an improvement in meat tenderness because calcium salts lead to an increase in the water content of the samples and increase calpain activation, with subsequent protein degradation and weakening of the myofibrillar structure. In a microstructure study, Sharedeh et al. [14] found that when meat was marinated it had swelling of the meat fibers and an increase in extracellular space, resulting in lower intercellular spaces in samples marinated with 2% salt than samples marinated with 0.9% salt. Nevertheless, our results showed no effect of marination on texture variables, which is in agreement with Kim, et al. [15], who stated that pressures above 200 MPa caused a decrease in water-holding capacity, resulting in a more compact structure that did not permit swelling.

## 5. Conclusions

Many significant interactions among factors were found in the present study. 

Under the applied conditions, it seems that the adiabatic increase in temperature and not the metmyoglobin content was responsible for the surface meat color, and in addition, the effects of pressure and marinade were so high that the effect of packaging was almost undetectable.

Independent of the storage type, pressurized treatments presented higher values for both L* and *h_ab_* than unpressurized treatments.

Independent of pressure application, the increase in L* and *h_ab_* with storage time was higher for marinated treatments than for unmarinated treatments. 

In unpressurized samples, marination provoked an increase in L*, a* and h_ab_ and a decrease in $C_{ab}^{*}$, whereas in pressurized samples, marination had no effect on color. 

Pressurized samples always showed higher values for shear stress (on average, 71% higher) than unpressurized samples. 

Further studies comparing different marinade ingredients or concentrations as well as different pressures are necessary in order to find an optimal combination which allows the meat conservation and tenderization without compromising the color.

## Figures and Tables

**Table 1:** The *p*-values of the effects of the studied factors (pressure application, packaging type, marination and storage time) on beef color variables.

	L*	a*	b*	$C_{ab}^{*}$	*h_ab_*	MMb
Pressure (P)	<0.001	<0.001	<0.001	0.011	<0.001	<0.001
Nano-packaging (N)	0.630	0.503	0.259	0.814	0.107	0.470
Marination (M)	<0.001	<0.001	0.140	<0.001	<0.001	<0.001
Storage time (T)	<0.001	<0.001	0.910	<0.001	<0.001	<0.001
P × N	<0.001	0.035	0.179	0.014	0.511	0.531
P × M	<0.001	<0.001	0.123	<0.001	<0.001	<0.001
P × T	<0.001	0.084	0.489	0.048	<0.001	<0.001
N × M	0.491	0.797	0.314	0.178	0.643	0.361
N × T	0.414	0.744	0.343	0.501	0.646	0.441
M × T	<0.001	<0.001	0.167	<0.001	<0.001	<0.001

MMb—metmyoglobin.

**Table 2:** The means and standard errors for beef color variables as a function of the studied factors (pressure application, packaging type, marinate immersion and storage time).

Treatment	Time, d	L*	a*	b*	h_ab_	C*	MMb %
C	0	40.3 cdx	15.4 ax	8.9 cx	30.2 cdx	17.8 abx	1.43 ax
	6	41.9 Dx	15.2 Ax	9.7 Cx	32.4 Dx	18.1 Ax	1.33 Ay
N	0	40.0 cdx	16.2 ax	9.6 bcx	30.9 cdx	18.9 ax	1.41 ax
	6	40.9 Dx	15.8 Ax	9.8 Cxx	32.0 Dx	18.6 Ax	1.29 Ay
M	0	41.5 cy	15.3 ax	10.3 bx	34.1 cy	18.5 ax	1.40 ax
	6	62.4 Bx	1.1 Cy	9.2 Cx	83.2 ABx	9.3 Dy	0.91 Cy
NM	0	39.3 dy	16.8 ax	8.8 cx	27.7 dy	19.1 ax	1.42 ax
	6	60.0 BCx	2.1 Cy	9.7 Cx	77.9 BCx	10.0 Dy	1.02 By
P	0	56.7 aby	8.5 bx	14.3 ax	59.6 aby	16.6 bx	0.96 bx
	6	59.2 Cx	4.4 By	14.1 ABx	72.8 Cx	14.7 BCy	0.82 Dy
PN	0	58.3 ay	8.0 bx	14.5 ax	61.3 ay	16.6 bx	1.03 bx
	6	60.8 BCx	4.0 By	15.2 Ax	75.4 Cx	15.7 Bx	0.83 Dy
PM	0	55.1 by	9.5 bx	13.6 ax	55.2 by	16.7 bx	1.07 bx
	6	67.4 Ax	0.5 Cy	13.3 Bx	87.7 Ax	13.3 Cy	0.92 Cy
PNM	0	58.2 ay	8.0 bx	14.4 ax	61.0 ay	16.5 bx	1.02 bx
	6	68.7 Ax	0.7 Cy	13.7 Bx	87.1 Ax	13.7 Cy	0.91 Cy
Standard error		1.03	0.594	0.267	2.145	0.295	0.023

a,b—different letters in a column imply significant differences between treatments at day zero of exposure time (*p* < 0.05); A,B—different letters in a column imply significant differences between treatments at six days of exposure time (*p* < 0.05). x,y—different letters in a column imply significant differences between storage times for a certain treatment (*p* < 0.05). C: Control (unpressurized-LPDE 0%-unmarinated); N: Unpressurized-LPDE 5%-unmarinated; M: Unpressurized-LPDE 0%-marinated; NM: Unpressurized-LPDE 5%-marinated; P: Pressurized-LPDE 0%-unmarinated; PN: Pressurized-LPDE 5%-unmarinated; PM: Pressurized-LPDE 0%-marinated; PNM: Pressurized-LPDE 5%-marinated.

**Table 3:** The *p*-values for the effects of the studied factors (pressure application, packaging type, marinade immersion and storage time) on beef shear stress and toughness.

	Shear Stress (N/cm^2^)	Toughness (N/cm^2^)
Pressure (P)	<0.001	<0.001
Nano-packaging (N)	0.575	0.852
Marination (M)	0.090	0.318
Storage time (T)	0.935	0.437
P × N	0.027	0.185
P × M	0.750	0.185
P × T	0.124	0.424
N × M	0.635	0.995
N × T	0.986	0.850
M × T	0.455	0.423

**Table 4:** The means and standard errors of beef shear stress and toughness as a function of treatment.

	Shear Stress (N/cm^2^)	Toughness (N/cm^2^)
C	40.5 ^bc^	13.0 ^c^
N	48.0 ^b^	16.1 ^bc^
M	38.7 ^c^	17.1 ^bc^
NM	43.6 ^bc^	17.8 ^abc^
P	78.9 ^a^	22.7 ^a^
PN	71.9 ^a^	20.1 ^ab^
PM	71.1 ^a^	21.1 ^ab^
PNM	70.7 ^a^	20.9 ^ab^
s.e.	2.21	0.67

a,b—different letters in a column imply significant differences between treatments (*p* < 0.05). C: Control (unpressurized-LPDE 0%-unmarinated); N: Unpressurized-LPDE 5%-unmarinated; M: Unpressurized-LPDE 0%-marinated; NM: Unpressurized-LPDE 5%-marinated; P: Pressurized-LPDE 0%-unmarinated; PN: Pressurized-LPDE 5%-unmarinated; PM: Pressurized-LPDE 0%-marinated; PNM: Pressurized-LPDE 5%-marinated.
